# Impact of prematurity and immigration on neonatal screening for sickle cell disease

**DOI:** 10.1371/journal.pone.0171604

**Published:** 2017-02-07

**Authors:** Ernesto Cortés-Castell, Antonio Palazón-Bru, Carolina Pla, Mercedes Goicoechea, María Mercedes Rizo-Baeza, Mercedes Juste, Vicente Francisco Gil-Guillén

**Affiliations:** 1 Department of Pharmacology, Pediatrics and Organic Chemistry, Miguel Hernández University, San Juan de Alicante, Alicante, Spain; 2 Department of Clinical Medicine, Miguel Hernández University, San Juan de Alicante, Alicante, Spain; 3 Department of Clinical Analysis, Alicante General University Hospital, Alicante, Alicante, Spain; 4 Center for Advanced Research in Public Health, Generalitat Valenciana, Valencia, Valencia, Spain; 5 Department of Nursing, University of Alicante, San Vicente del Raspeig, Alicante, Spain; Centre Hospitalier Universitaire Vaudois, FRANCE

## Abstract

**Background:**

Others have described a relationship between hemoglobin A levels and gestational age, gender and ethnicity. However, studies are needed to determine normal cut-off points considering these factors. To address this issue we designed a study to determine the percentiles of normality of neonatal hemoglobin A levels taking these factors into account.

**Methods:**

This cross-sectional study involved 16,025 samples for sickle cell disease screening in the province of Alicante, Spain, which has a high immigration rate. The primary variable was hemoglobin A, and the secondary variables were gender, gestational age (preterm and full term) and maternal origin (Spain, the rest of Europe, North Africa, Sub-Saharan Africa, Latin America and Asia). Percentiles of normality (1 and 99) were obtained by origin, gender and gestational age using quantile regression models and bootstrap samples. The association between these percentiles of normality and altered levels (≥1%) of hemoglobin E was analyzed. We obtained the percentiles of normality (1 and 99) for each maternal origin, gender and gestational age.

**Results:**

Of a total of 88 possible E carriers, 65 had above-normal hemoglobin A levels (74%). The levels of normality for hemoglobin A varied greatly according to the maternal origin and gestational age.

**Conclusion:**

With the levels of normality that we established it is possible to discard samples with unrecorded blood transfusions. Our methodology could be applied to other diseases in the neonatal screening.

## Introduction

Early detection of sickle cell disease and other hemoglobin disorders has traditionally only been included in newborn screening programs in geographical areas in which the population comprised ethnicities belonging to risk groups, such as in Jamaica or in the state of New York [1,2]. The great population movement and settlement in countries different to those of origin is resulting in the emergence of diseases in geographic areas where they historically did not exist [3,4]. The global incidence (or prevalence at birth) of hemoglobin disorders in 2008 was estimated at 228/10,000,000 births and in the US it was estimated at 139/10,000,000 [5,6]. In Spain the incidence is not high, but both the incidence and prevalence of abnormal hemoglobins is increasing as a result of immigration, especially immigrants of African origin, who have a high prevalence of hemoglobinopathies [7].

Complications of sickle cell disease can be delayed and mitigated with early detection [8–10], which explains the importance of early detection [11,12]. Consequently, neonatal screening for sickle cell anemia is being introduced progressively in Spain and other European countries, as well as elsewhere.

Since the introduction of neonatal screening for sickle cell disease, the incidence in different populations and the efficacy of early treatment have been reported, but normal values for the different neonatal hemoglobins according to variations in gestational age and ethnicity, in relation to the presence of abnormal hemoglobins, have not been established. The objectives of this study were to analyze the percentiles of normality for hemoglobin A in neonatal screening and their possible variation with prematurity and immigration. These percentiles can be used to establish cut-off points to facilitate the exclusion of samples outside the normal ranges, such as those with blood transfusions, that would require subsequent repetition.

## Materials and methods

### Study population

Newborns in the province of Alicante, which has a high percentages of newborns of immigrant mothers, according to data from 2010: 8.3% from the rest of Europe, 7.2% from Latin-America, 5.8% from North Africa, 1.1% from Asia, and 1.0% from sub-Saharan Africa [13].

### Design and participants

Cross-sectional observational study involving all newborns in the province of Alicante who underwent neonatal screening for sickle cell disease (n = 16,025) from March 2012 to February 2013 (heel prick taken at the first week of life). Prior informed consent was given by their parents or guardians (over 99% of newborns). We excluded any dried blood spots that did not meet the pre-analytical quality criteria [14], specimens that did not include all the study variables (gender, gestational age and maternal origin), specimens from newborns who were known to have undergone blood transfusion prior to sampling and anomalous samples, defined as those in which the presence of hemoglobins C, D, E or S was ≥1%.

### Variables and measures

The primary variable was the percentage of hemoglobin A. Secondary variables were gender, gestational age and place of maternal origin. The subgroups for each secondary variable were defined as: i) preterm (gestational age <37 weeks) [15]; ii) maternal origin, i.e. Spain, North Africa, sub-Saharan Africa, Latin-America, the rest of Europe and Asia; and iii) gender (male or female). All variables were obtained from the neonatal screening report.

### Laboratory tests

Detection and quantification of the different hemoglobins present in the newborn were performed with the Bio-Rad VARIANT nbs Sickle Cell Program kit based on cation-exchange high-performance liquid chromatography (HPLC) for separation and identification of hemoglobin F, A, C, D, E/A2 and S. Each hemoglobin type has a characteristic retention time, with the exception of E and A2 that elute in the same peak, therefore making them indistinguishable from each other. Hemoglobins were extracted from the blood spot paper into microplate wells and automatically injected into the chromatography system. Abs_415nm_ was measured in the different fractions eluted, minimizing background noise through the secondary wavelength at 690 nm. Absorbance data were recorded and analyzed using the VARIANT nbs Sickle Cell Program real-time chromatogram software, with summaries of the peaks identified, retention time and relative percentage. Samples were considered normal when not exceeding 1% in any of the abnormal hemoglobin peaks. FAES and FADC markers were introduced in each series analyzed [16].

### Statistical study

All analyses were performed with a 5% alpha level. The statistical software used was IBM SPSS Statistics 22 and R 2.13.2. The study variables were described using the standard methodology in health sciences (frequencies and percentages). One thousand bootstrap samples were obtained to estimate quantile regression models, using as dependent variables the 1^st^ percentile (P1) and the 99th percentile (P99) of the percentages of hemoglobin A, and as secondary variables gender, origin and prematurity. Based on the coefficients of the models we obtained 1,000 predictions for P1 and P99 in the twenty four subgroups [gender (2) · origin (6) · prematurity (2)]. In other words, we obtained the distribution of P1 and P99. Based on these values, confidence intervals (CI) were constructed using the percentile method. The estimations of the cut-off points (median of P1 and P99 for each subgroup) were represented in a Cartesian graph. Finally, the samples from children with altered levels of hemoglobin E were then analyzed based on the obtained cut-off points, calculating the percentage of newborns higher than the cut-off point (P99).

### Ethical considerations

Neonatal screening studies were approved by the Ethics Committee of the General Direction and Advanced Research in Public Health of the Valencian Community (code ACN), not requiring the informed consent of the newborn’s parent or guardian, in compliance with the current legislation in medical ethics. Moreover, the data, which are data from routine clinical practice in neonatal screening, were anonymized and encrypted, satisfying the data protection law.

## Results

Of the total of 16,025 newborns examined, 51.2% were boys (8,201) and 48.8% girls (7,824). One case of homozygous FSS was found in the total study sample, with maternal origin in sub-Saharan Africa, and no heterozygotes combined with any other abnormal hemoglobin of those studied. The number of abnormal samples from potential carriers was 47 FAC (0.29%); 12 FAD (0.07%); 88 possible FAE (0.55%) and 42 FAS (0.26%). Regarding gestational age, 8.4% (1,338) were preterm births. Concerning maternal origin, 77.1% (12,354) were from Spain; 8.0% (1,238) the rest of Europe; 6.2% (989) Latin-America; 6.4% (1,019) North Africa; 1.7% (278) Asia; and 0.6% (102) sub-Saharan Africa.

For males, Table 1 shows the means of the P1 and P99 of the percentage of hemoglobin A, with their 95% CI, in relation to maternal origin and gestational age (pre-term or full-term). For females, Table 2 shows these results.

**Table 1:** Atypical hemoglobin A values (%) according to maternal origin and gestational age for male gender.

	P1 (95% CI)		P99 (95% CI)	
Origin	< 37 w	≥ 37 w	< 37 w	≥ 37 w
Spain	4.2(3.7,4.7)	7.6(7.3,7.8)	37.5(31.8,46.9)	33.8(33.2,34.6)
Rest of Europe	4.2(3.5,5.1)	7.6(7.0,8.3)	36.0(30.2,45.0)	32.6(30.9,33.8)
Latin-America	4.2(3.5,4.9)	7.5(7.0,8.2)	40.2(33.6,49.2)	35.8(33.9,38.5)
North Africa	3.6(2.8,4.3)	6.9(6.6,7.3)	40.0(32.9,48.0)	35.3(33.2,37.5)
Asia	2.4(1.3,4.2)	5.5(5.0,7.6)	66.5(32.8,66.5)	59.4(32.8,68.4)
Sub-Saharan Africa	3.5(1.9,4.4)	6.9(5.4,7.5)	40.6(30.1,48.1)	36.5(29.5,39.7)

Abbreviations: w, weeks; P, percentile; CI, confidence interval.

**Table 2:** Atypical hemoglobin A values (%) according to maternal origin and gestational age for female gender.

	P1 (95% CI)		P99 (95% CI)	
Origin	< 37 w	≥ 37 w	< 37 w	≥ 37 w
Spain	5.0(4.6,5.5)	8.4(8.1,8.6)	38.6(32.6,47.5)	34.8(34.0,35.6)
Rest of Europe	4.9(4.4,5.9)	8.3(7.7,9.1)	37.5(31.2,45.8)	33.4(31.8,34.7)
Latin-America	5.0(4.4,5.7)	8.3(7.8,9.0)	41.2(34.5,49.5)	36.9(35.1,39.5)
North Africa	4.3(3.6,5.0)	7.6(7.1,8.2)	41.2(33.9,48.8)	36.3(34.5,38.2)
Asia	3.1(2.1,5.0)	6.3(5.5,8.3)	67.2(34.0,68.4)	60.3(34.2,69.5)
Sub-Saharan Africa	4.4(2.7,5.1)	7.7(6.1,8.4)	41.5(31.0,49.0)	37.7(31.1,40.4)

Abbreviations: w, weeks; P, percentile; CI, confidence interval.

**Fig 1:**
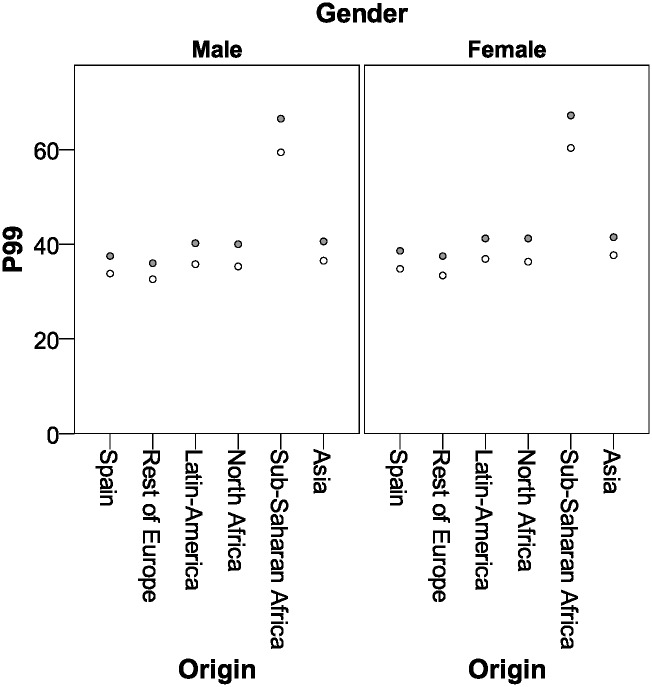
Atypical hemoglobin A values (P99 estimation in %) according to maternal origin, gestational age and gender. Grey, <37 weeks of gestational age; White, ≥37 weeks of gestational age.

**Fig 2:**
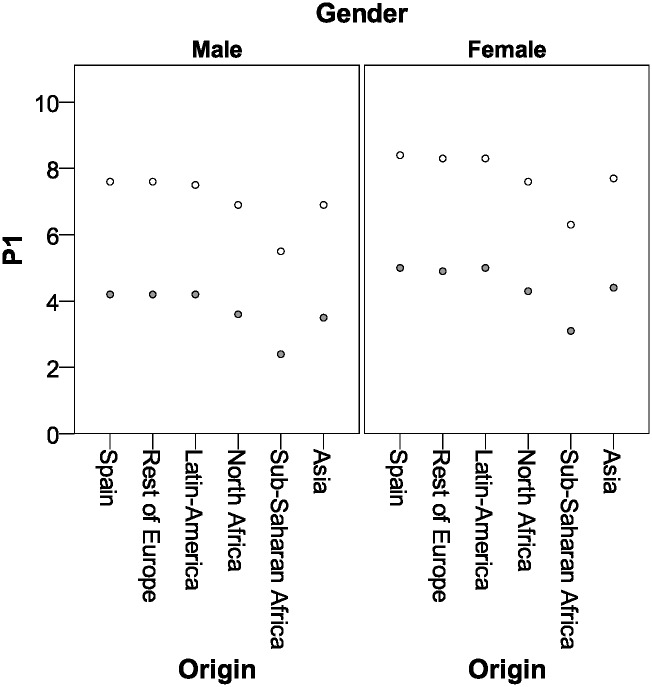
Atypical hemoglobin A values (P1 estimation in %) according to maternal origin, gestational age and gender. Grey, <37 weeks of gestational age; White, ≥37 weeks of gestational age.

Figs 1 and 2 show the estimation of the cut-off points for males and females, respectively, taking into account the origin and the gestational age.

From these percentiles we determined the percentiles in which newborns with an abnormal hemoglobin E were situated. The only cases with abnormal hemoglobin in which the presence of hemoglobin A was simultaneously greater than the estimation for P99 were 65 cases of suspected carriers of hemoglobin E. However, as indicated previously, the chromatographic E peak overlaps A2 and removing these 65 cases with atypically high values of A leaves 23 true cases of possible E carriers, with an incidence of 0.14%.

## Discussion

In this paper we calculated the percentiles of normality for the percentage of hemoglobin A considering gestational age, maternal origin and gender. The percentages of the different normal hemoglobins are influenced by gestational age, with this profile also being very different in infants than in older children and adults. With effect from birth, fetal hemoglobin decreases and hemoglobin A increases [17–19]. In newborn screening programs [13], sample collection within the first week of life is recommended, but older samples taken later are not rejected, showing that the percentiles of normality can also be used within an acceptable range of days.

Additionally, the percentages of the different hemoglobins varied depending on maternal origin. These differences can be explained when considering that closely related diseases, such as β-thalassemia and malaria, are endemic to areas where the highest persistent proportions of hemoglobin F have been found [20–23].

Regarding the presence of abnormal hemoglobins, we should highlight the situation with the apparent E carriers (n = 88), of whom 74% (n = 65) had much higher percentages of A than normal, raising the possibility of the E peak being masked by A2 caused by blood transfusions not recorded in the screening documents. After eliminating these samples as carriers, only 23 FAE remained. The only possibility of a neonatal sample having high levels of A is that the newborn has been subjected to one or more blood transfusions. These results demonstrate the value of using percentiles of normality to reject samples which could distort the results of neonatal screening.

### Strengths and limitations

The main strength of our study was that we obtained percentiles of normality in a large sample, demonstrating the feasibility of introducing reliable cut-off points that may enable automation of neonatal screening for sickle cell disease. These percentiles, although varying with gestational age, gender and ethnicity, perfectly discriminate incorrectly-taken samples, especially unreported transfusions.

Although very different risk factors were observed according to ethnic origin, further confirmatory studies over consecutive years should be undertaken in our environment. Regarding selection bias, a sample with a large number of children was analyzed over a specific period of one complete year. On the other hand, to minimize information bias, calibrated and validated equipment was used, together with a thorough review of the neonatal screening reports. Furthermore, the influence of thalassaemias (alpha and beta) on the level of hemoglobin A should have been reported. However, we have to take into account that these could not be diagnosed in the first week of life. On the other hand, unfortunately, for various reasons no data were gathered about the origin of the maternal grandparents. Finally, as these results can realistically only be extrapolated to the province of Alicante and the surrounding area, similar studies should be undertaken in other geographical areas, both in Spain and elsewhere.

## Implications to clinical practice

Our normal hemoglobin values enable the establishment of cut-off points relative to the mother's gender, age and origin, which may give greater sensitivity and specificity to the neonatal screening test for sickle-cell anemia. These values are adequate and, therefore, it is advisable to obtain them at all neonatal screening centers. Thus, our results and methodology are important in the field of public health and the organization of neonatal care.

## Conclusions

The cut-off points with the percentiles of normality of neonatal hemoglobin A were obtained taking into account maternal origin, gender and prematurity, which enables automation of the screening process and differentiation of newborns who have undergone a blood transfusion. The inclusion of these percentiles of normality could provide important savings in both cost and discomfort to the newborn and family by avoiding retesting many of the questionable samples. Finally, this methodology could help us develop neonatal screening protocols for other diseases to obtain cut-offs points using the bootstrap method and quantile regression models.

## Supporting information

S1 Dataset Raw data. (XLS)
